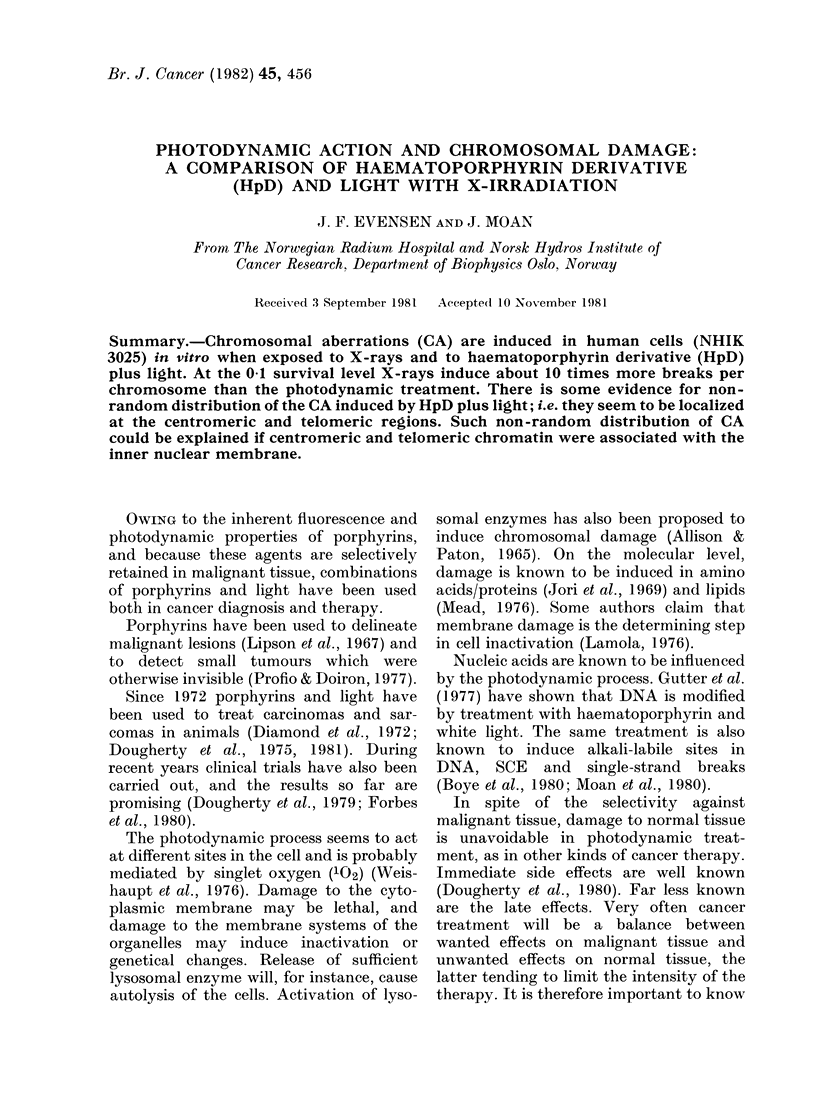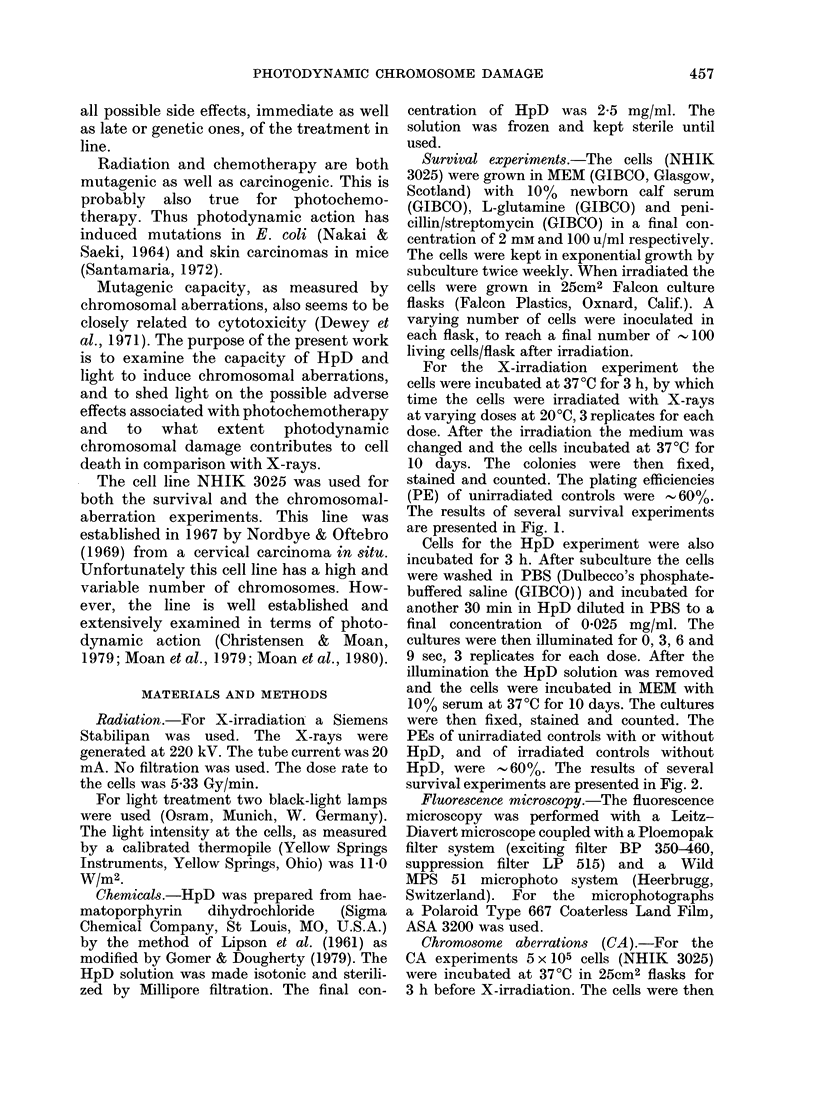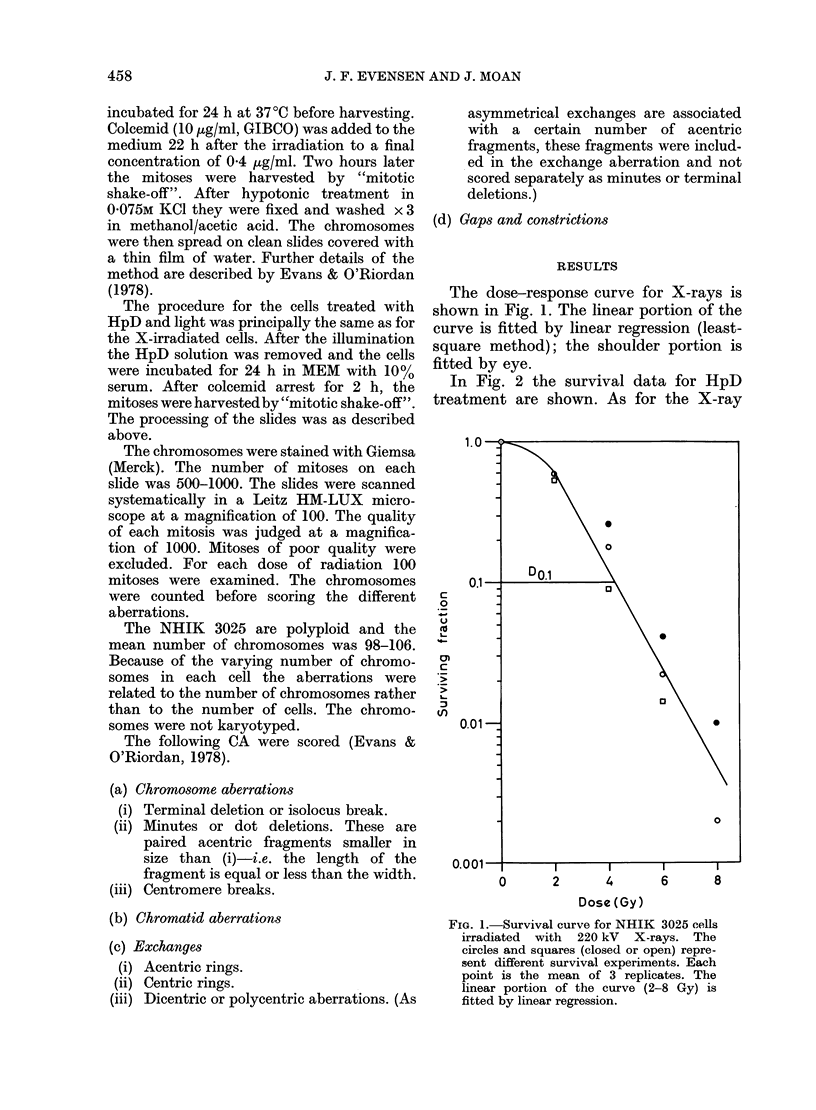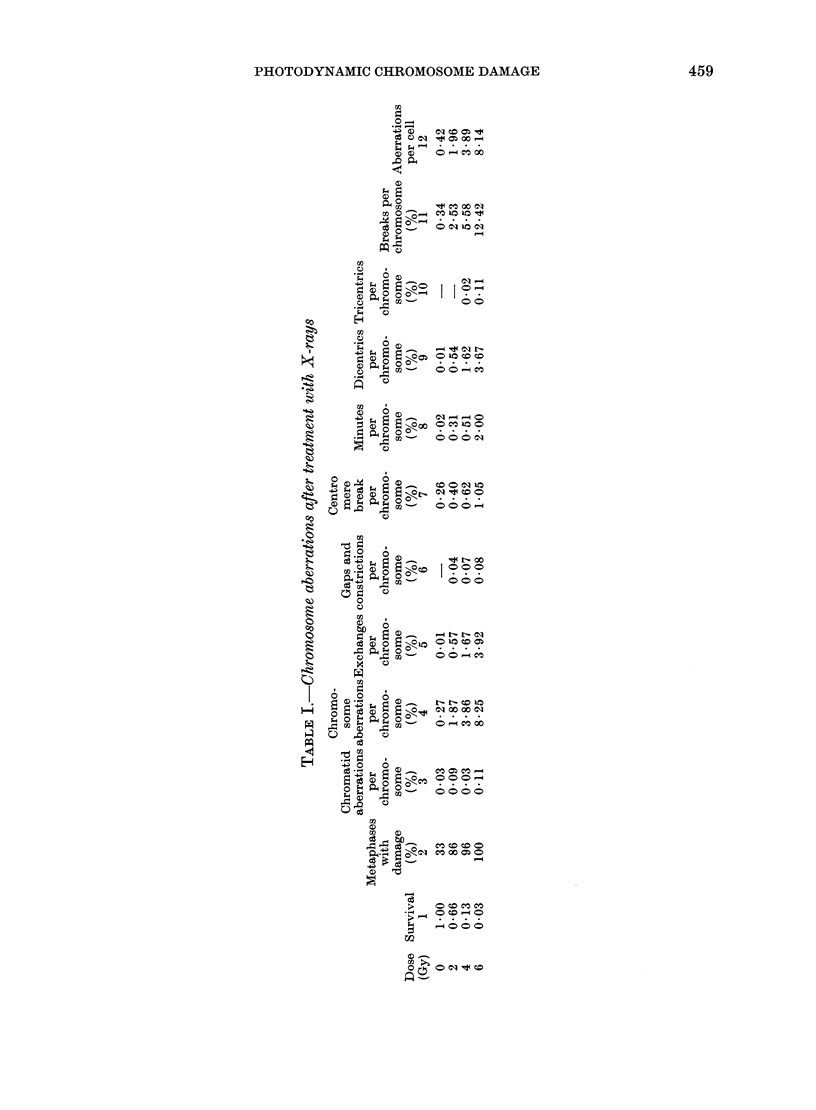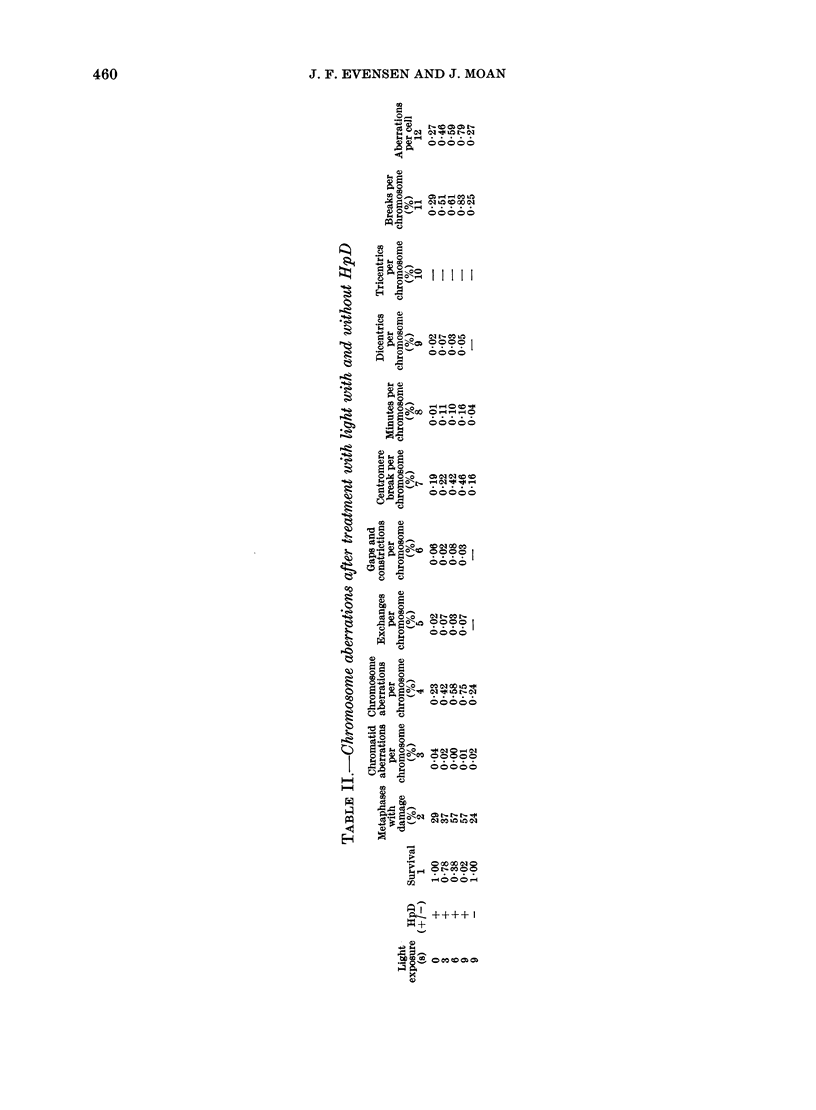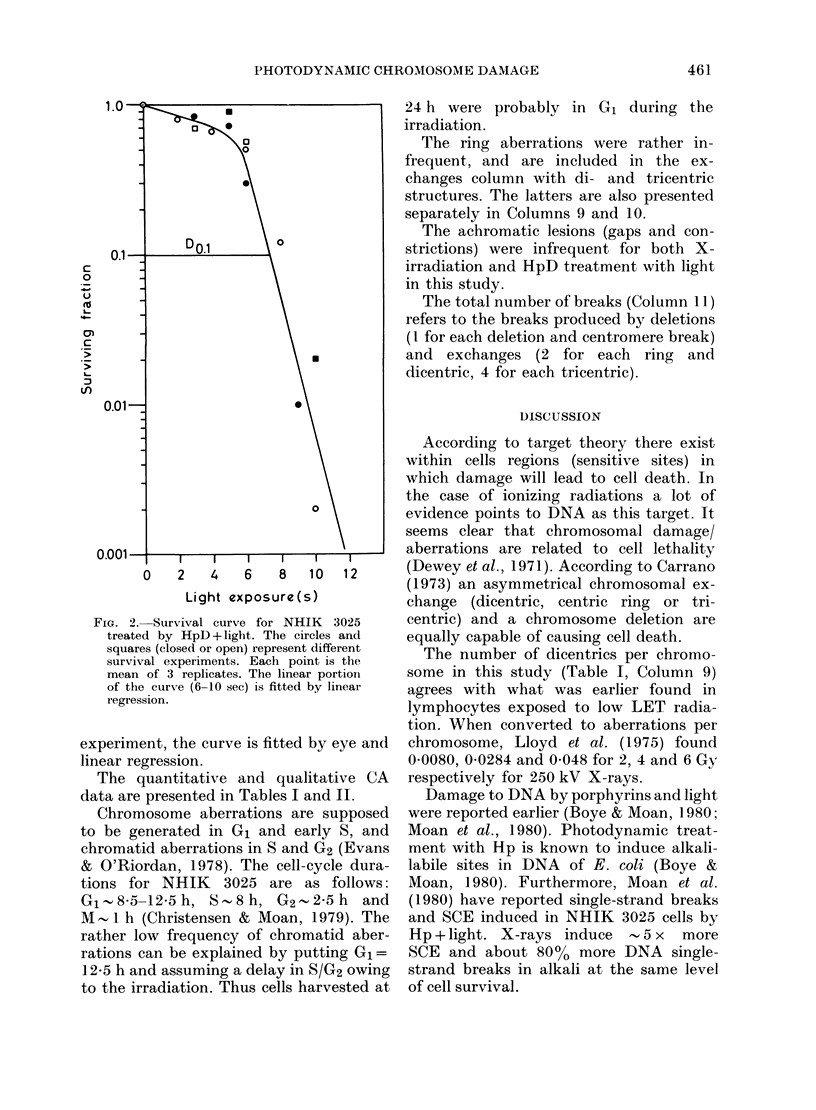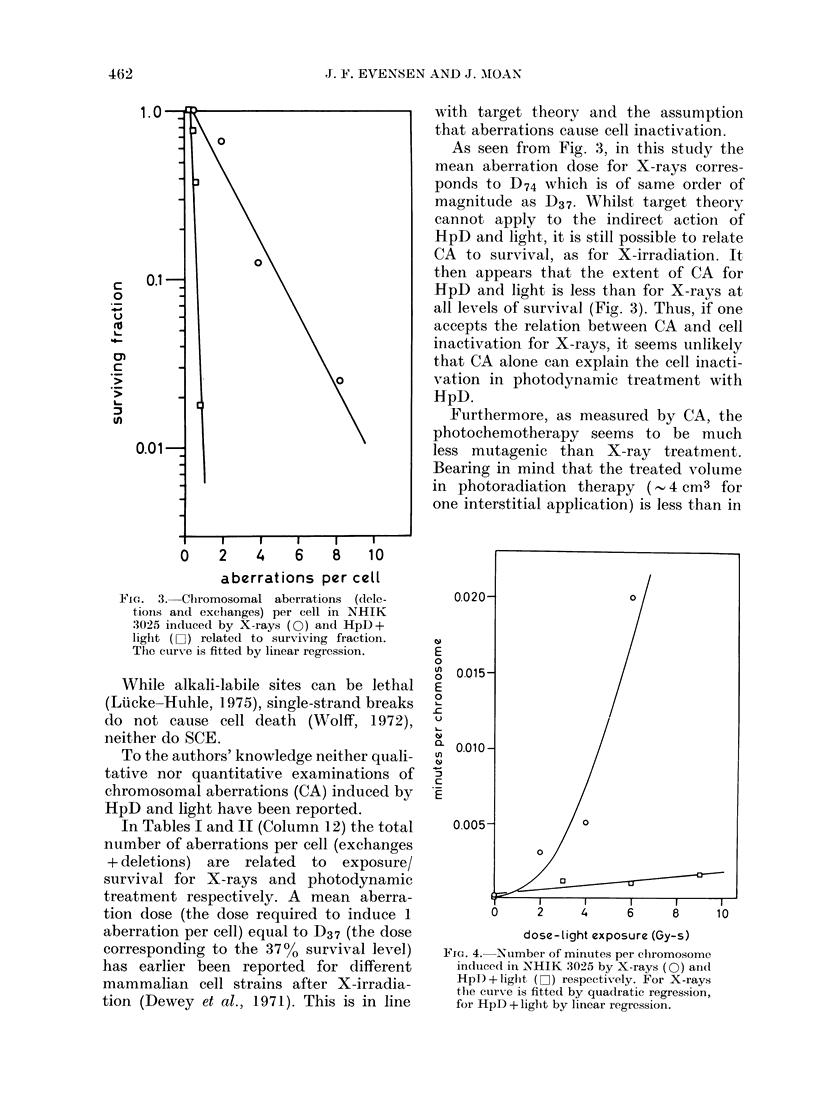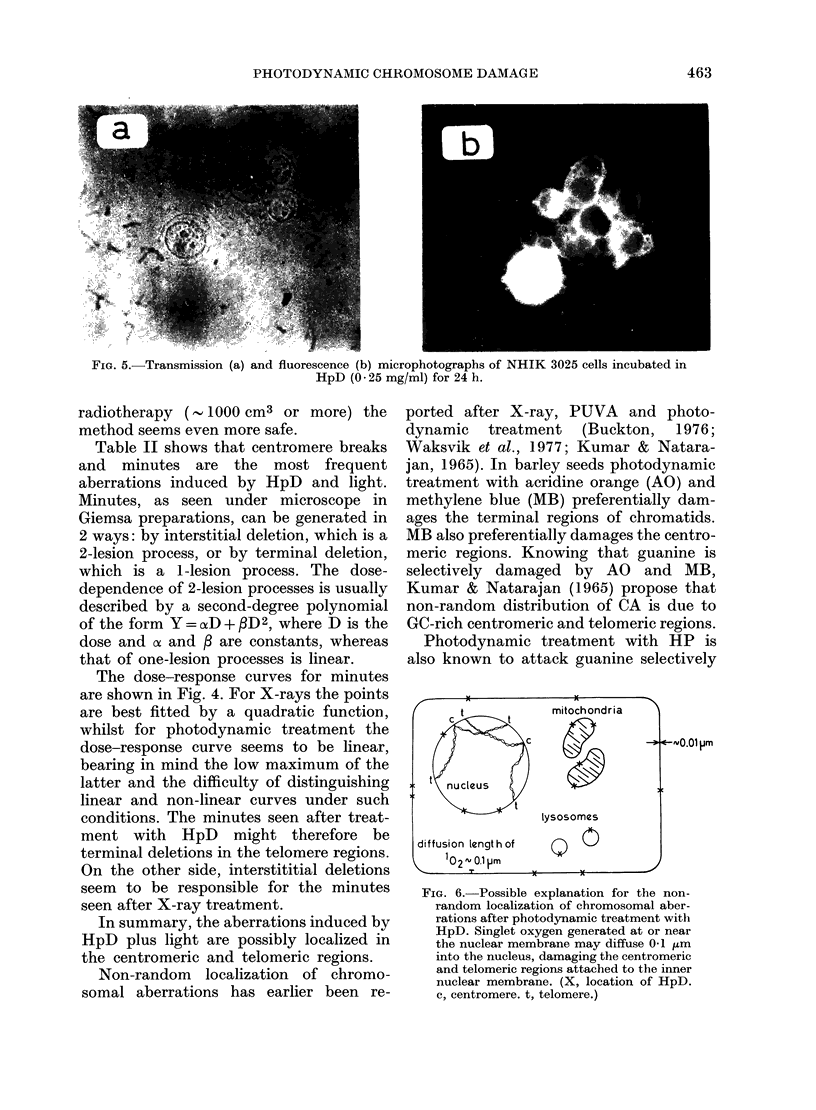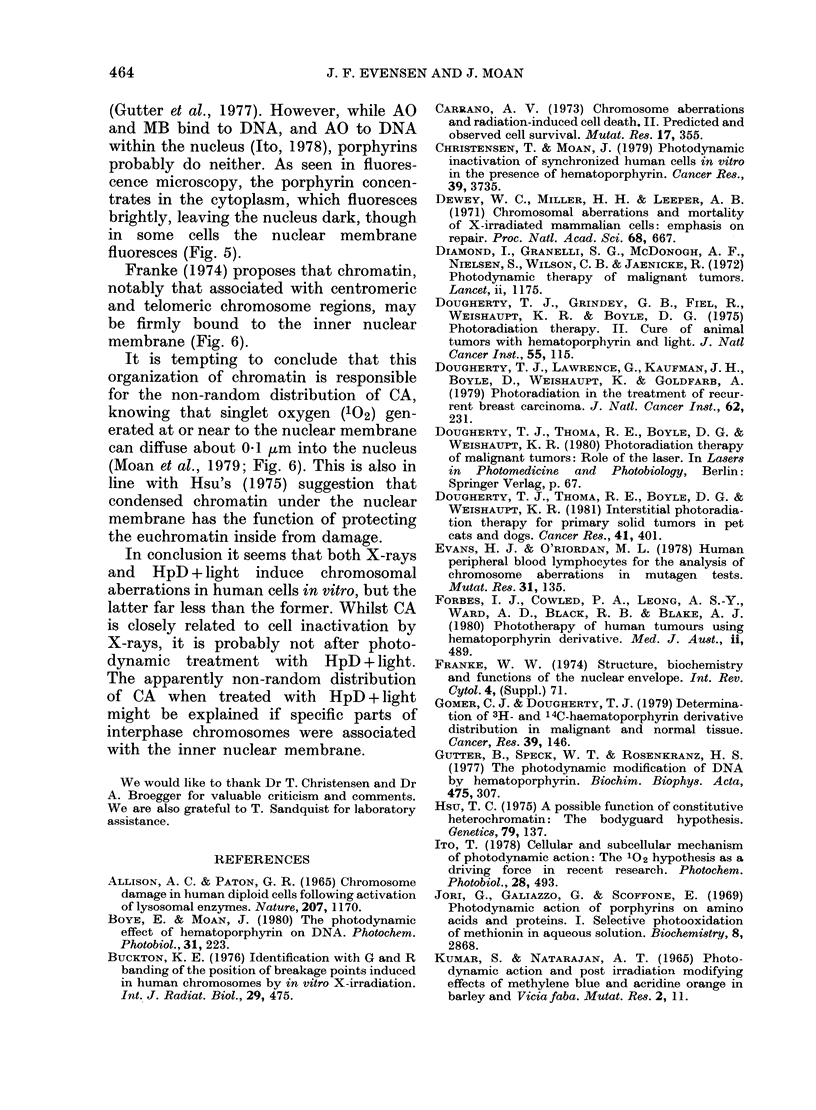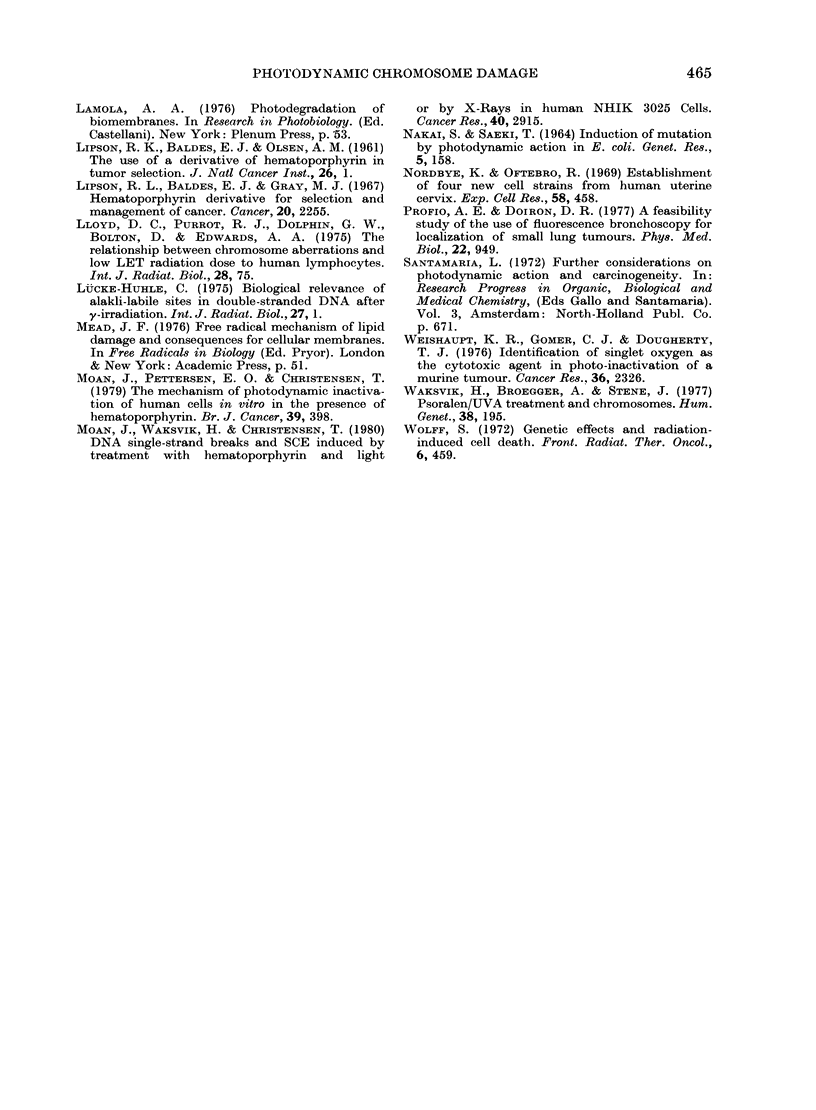# Photodynamic action and chromosomal damage: a comparison of haematoporphyrin derivative (HpD) and light with X-irradiation.

**DOI:** 10.1038/bjc.1982.74

**Published:** 1982-03

**Authors:** J. F. Evensen, J. Moan

## Abstract

**Images:**


*Br. J. Cancer* (1982) **45**, 456

### PHOTODYNAMIC ACTION AND CHROMOSOMAL DAMAGE: A COMPARISON OF HAEMATOPORPHYRIN DERIVATIVE (HpD) AND LIGHT WITH X-IRRADIATION

J. F. EVENSEN AND J. MOAN

*From The Norwegian Radium Hospital and Norsk Hydros Institute of Cancer Research, Department of Biophysics Oslo, Norway*

Received 3 September 1981 Accepted 10 November 1981

**Summary.—Chromosomal aberrations (CA) are induced in human cells (NHIK 3025) *in vitro* when exposed to X-rays and to haematoporphyrin derivative (HpD) plus light. At the 0·1 survival level X-rays induce about 10 times more breaks per chromosome than the photodynamic treatment. There is some evidence for non-random distribution of the CA induced by HpD plus light; *i.e.* they seem to be localized at the centromeric and telomeric regions. Such non-random distribution of CA could be explained if centromeric and telomeric chromatin were associated with the inner nuclear membrane.**

OWING to the inherent fluorescence and photodynamic properties of porphyrins, and because these agents are selectively retained in malignant tissue, combinations of porphyrins and light have been used both in cancer diagnosis and therapy.

Porphyrins have been used to delineate malignant lesions (Lipson *et al.*, 1967) and to detect small tumours which were otherwise invisible (Profio & Doiron, 1977).

Since 1972 porphyrins and light have been used to treat carcinomas and sarcomas in animals (Diamond *et al.*, 1972; Dougherty *et al.*, 1975, 1981). During recent years clinical trials have also been carried out, and the results so far are promising (Dougherty *et al.*, 1979; Forbes *et al.*, 1980).

The photodynamic process seems to act at different sites in the cell and is probably mediated by singlet oxygen ($^1O_2$) (Weishaupt *et al.*, 1976). Damage to the cytoplasmic membrane may be lethal, and damage to the membrane systems of the organelles may induce inactivation or genetical changes. Release of sufficient lysosomal enzyme will, for instance, cause autolysis of the cells. Activation of lysosomal enzymes has also been proposed to induce chromosomal damage (Allison & Paton, 1965). On the molecular level, damage is known to be induced in amino acids/proteins (Jori *et al.*, 1969) and lipids (Mead, 1976). Some authors claim that membrane damage is the determining step in cell inactivation (Lamola, 1976).

Nucleic acids are known to be influenced by the photodynamic process. Gutter *et al.* (1977) have shown that DNA is modified by treatment with haematoporphyrin and white light. The same treatment is also known to induce alkali-labile sites in DNA, SCE and single-strand breaks (Boye *et al.*, 1980; Moan *et al.*, 1980).

In spite of the selectivity against malignant tissue, damage to normal tissue is unavoidable in photodynamic treatment, as in other kinds of cancer therapy. Immediate side effects are well known (Dougherty *et al.*, 1980). Far less known are the late effects. Very often cancer treatment will be a balance between wanted effects on malignant tissue and unwanted effects on normal tissue, the latter tending to limit the intensity of the therapy. It is therefore important to know all possible side effects, immediate as well as late or genetic ones, of the treatment in line.

Radiation and chemotherapy are both mutagenic as well as carcinogenic. This is probably also true for photochemotherapy. Thus photodynamic action has induced mutations in *E. coli* (Nakai & Saeki, 1964) and skin carcinomas in mice (Santamaria, 1972).

Mutagenic capacity, as measured by chromosomal aberrations, also seems to be closely related to cytotoxicity (Dewey *et al.*, 1971). The purpose of the present work is to examine the capacity of HpD and light to induce chromosomal aberrations, and to shed light on the possible adverse effects associated with photochemotherapy and to what extent photodynamic chromosomal damage contributes to cell death in comparison with X-rays.

The cell line NHIK 3025 was used for both the survival and the chromosomal-aberration experiments. This line was established in 1967 by Nordbye & Oftebro (1969) from a cervical carcinoma *in situ*. Unfortunately this cell line has a high and variable number of chromosomes. However, the line is well established and extensively examined in terms of photodynamic action (Christensen & Moan, 1979; Moan *et al.*, 1979; Moan *et al.*, 1980).

### MATERIALS AND METHODS

*Radiation.*—For X-irradiation a Siemens Stabilipan was used. The X-rays were generated at 220 kV. The tube current was 20 mA. No filtration was used. The dose rate to the cells was 5·33 Gy/min.

For light treatment two black-light lamps were used (Osram, Munich, W. Germany). The light intensity at the cells, as measured by a calibrated thermopile (Yellow Springs Instruments, Yellow Springs, Ohio) was 11·0 $W/m^2$.

*Chemicals.*—HpD was prepared from haematoporphyrin dihydrochloride (Sigma Chemical Company, St Louis, MO, U.S.A.) by the method of Lipson *et al.* (1961) as modified by Gomer & Dougherty (1979). The HpD solution was made isotonic and sterilized by Millipore filtration. The final concentration of HpD was 2·5 mg/ml. The solution was frozen and kept sterile until used.

*Survival experiments.*—The cells (NHIK 3025) were grown in MEM (GIBCO, Glasgow, Scotland) with 10% newborn calf serum (GIBCO), L-glutamine (GIBCO) and penicillin/streptomycin (GIBCO) in a final concentration of 2 mM and 100 u/ml respectively. The cells were kept in exponential growth by subculture twice weekly. When irradiated the cells were grown in $25cm^2$ Falcon culture flasks (Falcon Plastics, Oxnard, Calif.). A varying number of cells were inoculated in each flask, to reach a final number of ~100 living cells/flask after irradiation.

For the X-irradiation experiment the cells were incubated at 37 °C for 3 h, by which time the cells were irradiated with X-rays at varying doses at 20 °C, 3 replicates for each dose. After the irradiation the medium was changed and the cells incubated at 37 °C for 10 days. The colonies were then fixed, stained and counted. The plating efficiencies (PE) of unirradiated controls were ~60%. The results of several survival experiments are presented in Fig. 1.

Cells for the HpD experiment were also incubated for 3 h. After subculture the cells were washed in PBS (Dulbecco's phosphate-buffered saline (GIBCO)) and incubated for another 30 min in HpD diluted in PBS to a final concentration of 0·025 mg/ml. The cultures were then illuminated for 0, 3, 6 and 9 sec, 3 replicates for each dose. After the illumination the HpD solution was removed and the cells were incubated in MEM with 10% serum at 37 °C for 10 days. The cultures were then fixed, stained and counted. The PEs of unirradiated controls with or without HpD, and of irradiated controls without HpD, were ~60%. The results of several survival experiments are presented in Fig. 2.

*Fluorescence microscopy.*—The fluorescence microscopy was performed with a Leitz–Diavert microscope coupled with a Ploemopak filter system (exciting filter BP 350–460, suppression filter LP 515) and a Wild MPS 51 microphoto system (Heerbrugg, Switzerland). For the microphotographs a Polaroid Type 667 Coaterless Land Film, ASA 3200 was used.

*Chromosome aberrations (CA).*—For the CA experiments $5 \times 10^5$ cells (NHIK 3025) were incubated at 37 °C in $25cm^2$ flasks for 3 h before X-irradiation. The cells were then incubated for 24 h at 37 °C before harvesting. Colcemid (10 $\mu$g/ml, GIBCO) was added to the medium 22 h after the irradiation to a final concentration of 0·4 $\mu$g/ml. Two hours later the mitoses were harvested by "mitotic shake-off". After hypotonic treatment in 0·075M KCl they were fixed and washed $\times 3$ in methanol/acetic acid. The chromosomes were then spread on clean slides covered with a thin film of water. Further details of the method are described by Evans & O'Riordan (1978).

The procedure for the cells treated with HpD and light was principally the same as for the X-irradiated cells. After the illumination the HpD solution was removed and the cells were incubated for 24 h in MEM with 10% serum. After colcemid arrest for 2 h, the mitoses were harvested by "mitotic shake-off". The processing of the slides was as described above.

The chromosomes were stained with Giemsa (Merck). The number of mitoses on each slide was 500–1000. The slides were scanned systematically in a Leitz HM-LUX microscope at a magnification of 100. The quality of each mitosis was judged at a magnification of 1000. Mitoses of poor quality were excluded. For each dose of radiation 100 mitoses were examined. The chromosomes were counted before scoring the different aberrations.

The NHIK 3025 are polyploid and the mean number of chromosomes was 98–106. Because of the varying number of chromosomes in each cell the aberrations were related to the number of chromosomes rather than to the number of cells. The chromosomes were not karyotyped.

The following CA were scored (Evans & O'Riordan, 1978).

(a) *Chromosome aberrations*

(i) Terminal deletion or isolocus break.
(ii) Minutes or dot deletions. These are paired acentric fragments smaller in size than (i)—*i.e.* the length of the fragment is equal or less than the width.
(iii) Centromere breaks.

(b) *Chromatid aberrations*

(c) *Exchanges*

(i) Acentric rings.
(ii) Centric rings.
(iii) Dicentric or polycentric aberrations. (As asymmetrical exchanges are associated with a certain number of acentric fragments, these fragments were included in the exchange aberration and not scored separately as minutes or terminal deletions.)

(d) *Gaps and constrictions*

### RESULTS

The dose–response curve for X-rays is shown in Fig. 1. The linear portion of the curve is fitted by linear regression (least-square method); the shoulder portion is fitted by eye.

In Fig. 2 the survival data for HpD treatment are shown. As for the X-ray

FIG. 1.—Survival curve for NHIK 3025 cells irradiated with 220 kV X-rays. The circles and squares (closed or open) represent different survival experiments. Each point is the mean of 3 replicates. The linear portion of the curve (2–8 Gy) is fitted by linear regression.

TABLE I.—*Chromosome aberrations after treatment with X-rays*

| Dose (Gy) | Survival 1 | Metaphases with damage (%) 2 | Chromatid aberrations per chromosome (%) 3 | Chromosome aberrations per chromosome (%) 4 | Exchanges per chromosome (%) 5 | Gaps and constrictions per chromosome (%) 6 | Centro mere break per chromosome (%) 7 | Minutes per chromosome (%) 8 | Dicentrics per chromosome (%) 9 | Tricentrics per chromosome (%) 10 | Breaks per chromosome (%) 11 | Aberrations per cell 12 |
|---|---|---|---|---|---|---|---|---|---|---|---|---|
| 0 | 1·00 | 33 | 0·03 | 0·27 | 0·01 | — | 0·26 | 0·02 | 0·01 | — | 0·34 | 0·42 |
| 2 | 0·66 | 86 | 0·09 | 1·87 | 0·57 | 0·04 | 0·40 | 0·31 | 0·54 | — | 2·53 | 1·96 |
| 4 | 0·13 | 96 | 0·03 | 3·86 | 1·67 | 0·07 | 0·62 | 0·51 | 1·62 | 0·02 | 5·58 | 3·89 |
| 6 | 0·03 | 100 | 0·11 | 8·25 | 3·92 | 0·08 | 1·05 | 2·00 | 3·67 | 0·11 | 12·42 | 8·14 |

TABLE II.—*Chromosome aberrations after treatment with light with and without HpD*

| Light exposure (s) | HpD (+/−) | Survival 1 | Metaphases with damage (%) 2 | Chromatid aberrations per chromosome (%) 3 | Chromosome aberrations per chromosome (%) 4 | Exchanges per chromosome (%) 5 | Gaps and constrictions per chromosome (%) 6 | Centromere break per chromosome (%) 7 | Minutes per chromosome (%) 8 | Dicentrics per chromosome (%) 9 | Tricentrics per chromosome (%) 10 | Breaks per chromosome (%) 11 | Aberrations per cell 12 |
|---|---|---|---|---|---|---|---|---|---|---|---|---|---|
| 0 | + | 1·00 | 29 | 0·04 | 0·23 | 0·02 | 0·06 | 0·19 | 0·01 | 0·02 | — | 0·29 | 0·27 |
| 3 | + | 0·78 | 37 | 0·02 | 0·42 | 0·07 | 0·02 | 0·22 | 0·11 | 0·07 | — | 0·51 | 0·46 |
| 6 | + | 0·38 | 57 | 0·00 | 0·58 | 0·03 | 0·08 | 0·42 | 0·10 | 0·03 | — | 0·61 | 0·59 |
| 9 | + | 0·02 | 57 | 0·01 | 0·75 | 0·07 | 0·03 | 0·46 | 0·16 | 0·05 | — | 0·83 | 0·79 |
| 9 | − | 1·00 | 24 | 0·02 | 0·24 | — | — | 0·16 | 0·04 | — | — | 0·25 | 0·27 |

FIG. 2.—Survival curve for NHIK 3025 treated by HpD + light. The circles and squares (closed or open) represent different survival experiments. Each point is the mean of 3 replicates. The linear portion of the curve (6–10 sec) is fitted by linear regression.

experiment, the curve is fitted by eye and linear regression.

The quantitative and qualitative CA data are presented in Tables I and II.

Chromosome aberrations are supposed to be generated in $G_1$ and early S, and chromatid aberrations in S and $G_2$ (Evans & O'Riordan, 1978). The cell-cycle durations for NHIK 3025 are as follows: $G_1 \sim 8{\cdot}5$–$12{\cdot}5$ h, $S \sim 8$ h, $G_2 \sim 2{\cdot}5$ h and $M \sim 1$ h (Christensen & Moan, 1979). The rather low frequency of chromatid aberrations can be explained by putting $G_1 = 12{\cdot}5$ h and assuming a delay in S/$G_2$ owing to the irradiation. Thus cells harvested at 24 h were probably in $G_1$ during the irradiation.

The ring aberrations were rather infrequent, and are included in the exchanges column with di- and tricentric structures. The latters are also presented separately in Columns 9 and 10.

The achromatic lesions (gaps and constrictions) were infrequent for both X-irradiation and HpD treatment with light in this study.

The total number of breaks (Column 11) refers to the breaks produced by deletions (1 for each deletion and centromere break) and exchanges (2 for each ring and dicentric, 4 for each tricentric).

### DISCUSSION

According to target theory there exist within cells regions (sensitive sites) in which damage will lead to cell death. In the case of ionizing radiations a lot of evidence points to DNA as this target. It seems clear that chromosomal damage/aberrations are related to cell lethality (Dewey *et al.*, 1971). According to Carrano (1973) an asymmetrical chromosomal exchange (dicentric, centric ring or tricentric) and a chromosome deletion are equally capable of causing cell death.

The number of dicentrics per chromosome in this study (Table I, Column 9) agrees with what was earlier found in lymphocytes exposed to low LET radiation. When converted to aberrations per chromosome, Lloyd *et al.* (1975) found 0·0080, 0·0284 and 0·048 for 2, 4 and 6 Gy respectively for 250 kV X-rays.

Damage to DNA by porphyrins and light were reported earlier (Boye & Moan, 1980; Moan *et al.*, 1980). Photodynamic treatment with Hp is known to induce alkali-labile sites in DNA of *E. coli* (Boye & Moan, 1980). Furthermore, Moan *et al.* (1980) have reported single-strand breaks and SCE induced in NHIK 3025 cells by Hp + light. X-rays induce $\sim 5\times$ more SCE and about 80% more DNA single-strand breaks in alkali at the same level of cell survival.

FIG. 3.—Chromosomal aberrations (deletions and exchanges) per cell in NHIK 3025 induced by X-rays (○) and HpD+light (□) related to surviving fraction. The curve is fitted by linear regression.

While alkali-labile sites can be lethal (Lücke-Huhle, 1975), single-strand breaks do not cause cell death (Wolff, 1972), neither do SCE.

To the authors' knowledge neither qualitative nor quantitative examinations of chromosomal aberrations (CA) induced by HpD and light have been reported.

In Tables I and II (Column 12) the total number of aberrations per cell (exchanges + deletions) are related to exposure/survival for X-rays and photodynamic treatment respectively. A mean aberration dose (the dose required to induce 1 aberration per cell) equal to $D_{37}$ (the dose corresponding to the 37% survival level) has earlier been reported for different mammalian cell strains after X-irradiation (Dewey *et al.*, 1971). This is in line with target theory and the assumption that aberrations cause cell inactivation.

As seen from Fig. 3, in this study the mean aberration dose for X-rays corresponds to $D_{74}$ which is of same order of magnitude as $D_{37}$. Whilst target theory cannot apply to the indirect action of HpD and light, it is still possible to relate CA to survival, as for X-irradiation. It then appears that the extent of CA for HpD and light is less than for X-rays at all levels of survival (Fig. 3). Thus, if one accepts the relation between CA and cell inactivation for X-rays, it seems unlikely that CA alone can explain the cell inactivation in photodynamic treatment with HpD.

Furthermore, as measured by CA, the photochemotherapy seems to be much less mutagenic than X-ray treatment. Bearing in mind that the treated volume in photoradiation therapy (~4 $cm^3$ for one interstitial application) is less than in

FIG. 4.—Number of minutes per chromosome induced in NHIK 3025 by X-rays (○) and HpD+light (□) respectively. For X-rays the curve is fitted by quadratic regression, for HpD+light by linear regression.

FIG. 5.—Transmission (a) and fluorescence (b) microphotographs of NHIK 3025 cells incubated in HpD (0·25 mg/ml) for 24 h.

radiotherapy (~1000 $cm^3$ or more) the method seems even more safe.

Table II shows that centromere breaks and minutes are the most frequent aberrations induced by HpD and light. Minutes, as seen under microscope in Giemsa preparations, can be generated in 2 ways: by interstitial deletion, which is a 2-lesion process, or by terminal deletion, which is a 1-lesion process. The dose-dependence of 2-lesion processes is usually described by a second-degree polynomial of the form $Y = \alpha D + \beta D^2$, where D is the dose and $\alpha$ and $\beta$ are constants, whereas that of one-lesion processes is linear.

The dose–response curves for minutes are shown in Fig. 4. For X-rays the points are best fitted by a quadratic function, whilst for photodynamic treatment the dose–response curve seems to be linear, bearing in mind the low maximum of the latter and the difficulty of distinguishing linear and non-linear curves under such conditions. The minutes seen after treatment with HpD might therefore be terminal deletions in the telomere regions. On the other side, interstititial deletions seem to be responsible for the minutes seen after X-ray treatment.

In summary, the aberrations induced by HpD plus light are possibly localized in the centromeric and telomeric regions.

Non-random localization of chromosomal aberrations has earlier been reported after X-ray, PUVA and photodynamic treatment (Buckton, 1976; Waksvik *et al.*, 1977; Kumar & Natarajan, 1965). In barley seeds photodynamic treatment with acridine orange (AO) and methylene blue (MB) preferentially damages the terminal regions of chromatids. MB also preferentially damages the centromeric regions. Knowing that guanine is selectively damaged by AO and MB, Kumar & Natarajan (1965) propose that non-random distribution of CA is due to GC-rich centromeric and telomeric regions.

Photodynamic treatment with HP is also known to attack guanine selectively

FIG. 6.—Possible explanation for the non-random localization of chromosomal aberrations after photodynamic treatment with HpD. Singlet oxygen generated at or near the nuclear membrane may diffuse 0·1 $\mu$m into the nucleus, damaging the centromeric and telomeric regions attached to the inner nuclear membrane. (X, location of HpD. c, centromere. t, telomere.)

(Gutter *et al.*, 1977). However, while AO and MB bind to DNA, and AO to DNA within the nucleus (Ito, 1978), porphyrins probably do neither. As seen in fluorescence microscopy, the porphyrin concentrates in the cytoplasm, which fluoresces brightly, leaving the nucleus dark, though in some cells the nuclear membrane fluoresces (Fig. 5).

Franke (1974) proposes that chromatin, notably that associated with centromeric and telomeric chromosome regions, may be firmly bound to the inner nuclear membrane (Fig. 6).

It is tempting to conclude that this organization of chromatin is responsible for the non-random distribution of CA, knowing that singlet oxygen ($^1O_2$) generated at or near to the nuclear membrane can diffuse about 0·1 $\mu$m into the nucleus (Moan *et al.*, 1979; Fig. 6). This is also in line with Hsu's (1975) suggestion that condensed chromatin under the nuclear membrane has the function of protecting the euchromatin inside from damage.

In conclusion it seems that both X-rays and HpD+light induce chromosomal aberrations in human cells *in vitro*, but the latter far less than the former. Whilst CA is closely related to cell inactivation by X-rays, it is probably not after photodynamic treatment with HpD+light. The apparently non-random distribution of CA when treated with HpD+light might be explained if specific parts of interphase chromosomes were associated with the inner nuclear membrane.

We would like to thank Dr T. Christensen and Dr A. Broegger for valuable criticism and comments. We are also grateful to T. Sandquist for laboratory assistance.

### REFERENCES

Allison, A. C. & Paton, G. R. (1965) Chromosome damage in human diploid cells following activation of lysosomal enzymes. *Nature*, **207**, 1170.

Boye, E. & Moan, J. (1980) The photodynamic effect of hematoporphyrin on DNA. *Photochem. Photobiol.*, **31**, 223.

Buckton, K. E. (1976) Identification with G and R banding of the position of breakage points induced in human chromosomes by *in vitro* X-irradiation. *Int. J. Radiat. Biol.*, **29**, 475.

Carrano, A. V. (1973) Chromosome aberrations and radiation-induced cell death. II. Predicted and observed cell survival. *Mutat. Res.* **17**, 355.

Christensen, T. & Moan, J. (1979) Photodynamic inactivation of synchronized human cells *in vitro* in the presence of hematoporphyrin. *Cancer Res.*, **39**, 3735.

Dewey, W. C., Miller, H. H. & Leeper, A. B. (1971) Chromosomal aberrations and mortality of X-irradiated mammalian cells: emphasis on repair. *Proc. Natl. Acad. Sci.* **68**, 667.

Diamond, I., Granelli, S. G., McDonogh, A. F., Nielsen, S., Wilson, C. B. & Jaenicke, R. (1972) Photodynamic therapy of malignant tumors. *Lancet*, ii, 1175.

Dougherty, T. J., Grindey, G. B., Fiel, R., Weishaupt, K. R. & Boyle, D. G. (1975) Photoradiation therapy. II. Cure of animal tumors with hematoporphyrin and light. *J. Natl Cancer Inst.*, **55**, 115.

Dougherty, T. J., Lawrence, G., Kaufman, J. H., Boyle, D., Weishaupt, K. & Goldfarb, A. (1979) Photoradiation in the treatment of recurrent breast carcinoma. *J. Natl. Cancer Inst.*, **62**, 231.

Dougherty, T. J., Thoma, R. E., Boyle, D. G. & Weishaupt, K. R. (1980) Photoradiation therapy of malignant tumors: Role of the laser. In *Lasers in Photomedicine and Photobiology*, Berlin: Springer Verlag, p. 67.

Dougherty, T. J., Thoma, R. E., Boyle, D. G. & Weishaupt, K. R. (1981) Interstitial photoradiation therapy for primary solid tumors in pet cats and dogs. *Cancer Res.*, **41**, 401.

Evans, H. J. & O'Riordan, M. L. (1978) Human peripheral blood lymphocytes for the analysis of chromosome aberrations in mutagen tests. *Mutat. Res.* **31**, 135.

Forbes, I. J., Cowled, P. A., Leong, A. S.-Y., Ward, A. D., Black, R. B. & Blake, A. J. (1980) Phototherapy of human tumours using hematoporphyrin derivative. *Med. J. Aust.*, **ii**, 489.

Franke, W. W. (1974) Structure, biochemistry and functions of the nuclear envelope. *Int. Rev. Cytol.* **4**, (Suppl.) 71.

Gomer, C. J. & Dougherty, T. J. (1979) Determination of $^3$H- and $^{14}$C-haematoporphyrin derivative distribution in malignant and normal tissue. *Cancer, Res.* **39**, 146.

Gutter, B., Speck, W. T. & Rosenkranz, H. S. (1977) The photodynamic modification of DNA by hematoporphyrin. *Biochim. Biophys. Acta*, **475**, 307.

Hsu, T. C. (1975) A possible function of constitutive heterochromatin: The bodyguard hypothesis. *Genetics*, **79**, 137.

Ito, T. (1978) Cellular and subcellular mechanism of photodynamic action: The $^1O_2$ hypothesis as a driving force in recent research. *Photochem. Photobiol.*, **28**, 493.

Jori, G., Galiazzo, G. & Scoffone, E. (1969) Photodynamic action of porphyrins on amino acids and proteins. I. Selective photooxidation of methionin in aqueous solution. *Biochemistry*, **8**, 2868.

Kumar, S. & Natarajan, A. T. (1965) Photodynamic action and post irradiation modifying effects of methylene blue and acridine orange in barley and *Vicia faba*. *Mutat. Res.* **2**, 11.

LAMOLA, A. A. (1976) Photodegradation of biomembranes. In *Research in Photobiology*. (Ed. Castellani). New York: Plenum Press, p. 53.

LIPSON, R. K., BALDES, E. J. & OLSEN, A. M. (1961) The use of a derivative of hematoporphyrin in tumor selection. *J. Natl Cancer Inst.*, **26**, 1.

LIPSON, R. L., BALDES, E. J. & GRAY, M. J. (1967) Hematoporphyrin derivative for selection and management of cancer. *Cancer*, **20**, 2255.

LLOYD, D. C., PURROT, R. J., DOLPHIN, G. W., BOLTON, D. & EDWARDS, A. A. (1975) The relationship between chromosome aberrations and low LET radiation dose to human lymphocytes. *Int. J. Radiat. Biol.*, **28**, 75.

LÜCKE-HUHLE, C. (1975) Biological relevance of alakli-labile sites in double-stranded DNA after $\gamma$-irradiation. *Int. J. Radiat. Biol.*, **27**, 1.

MEAD, J. F. (1976) Free radical mechanism of lipid damage and consequences for cellular membranes. In *Free Radicals in Biology* (Ed. Pryor). London & New York: Academic Press, p. 51.

MOAN, J., PETTERSEN, E. O. & CHRISTENSEN, T. (1979) The mechanism of photodynamic inactivation of human cells *in vitro* in the presence of hematoporphyrin. *Br. J. Cancer*, **39**, 398.

MOAN, J., WAKSVIK, H. & CHRISTENSEN, T. (1980) DNA single-strand breaks and SCE induced by treatment with hematoporphyrin and light or by X-Rays in human NHIK 3025 Cells. *Cancer Res.*, **40**, 2915.

NAKAI, S. & SAEKI, T. (1964) Induction of mutation by photodynamic action in *E. coli*. *Genet. Res.*, **5**, 158.

NORDBYE, K. & OFTEBRO, R. (1969) Establishment of four new cell strains from human uterine cervix. *Exp. Cell Res.*, **58**, 458.

PROFIO, A. E. & DOIRON, D. R. (1977) A feasibility study of the use of fluorescence bronchoscopy for localization of small lung tumours. *Phys. Med. Biol.*, **22**, 949.

SANTAMARIA, L. (1972) Further considerations on photodynamic action and carcinogeneity. In: *Research Progress in Organic, Biological and Medical Chemistry*, (Eds Gallo and Santamaria). Vol. 3, Amsterdam: North-Holland Publ. Co. p. 671.

WEISHAUPT, K. R., GOMER, C. J. & DOUGHERTY, T. J. (1976) Identification of singlet oxygen as the cytotoxic agent in photo-inactivation of a murine tumour. *Cancer Res.*, **36**, 2326.

WAKSVIK, H., BROEGGER, A. & STENE, J. (1977) Psoralen/UVA treatment and chromosomes. *Hum. Genet.*, **38**, 195.

WOLFF, S. (1972) Genetic effects and radiation-induced cell death. *Front. Radiat. Ther. Oncol.*, **6**, 459.